# Translating clinical research of Molecular Biology into a personalized, multidisciplinary approach of colorectal cancer patients

**Published:** 2014-03-25

**Authors:** V Strambu, D Garofil, F Pop, P Radu, M Bratucu, F Popa

**Affiliations:** * Department of General Surgery, “Carol Davila” Clinical Nephrology Hospital, Bucharest, Romania; ** Department of General Surgery, “Sf. Pantelimon” Clinical Emergency Hospital, Bucharest, Romania; *** Department of Pathology, “Carol Davila” Clinical Nephrology Hospital, Bucharest, Romania

**Keywords:** colorectal, cancer, microsatellite, instability, KRAS

## Abstract

Abstract

Although multimodal treatment has brought important benefit, there is still great heterogeneity regarding the indication and response to chemotherapy in Stage II and III, and individual variations related to both overall survival and toxicity of new therapies in metastatic disease or tumor relapse. Recent research in molecular biology led to the development of a large scale of genetic biomarkers, but their clinical use is not concordant with the high expectations.

The Aim of this review is to identify and discuss the molecular markers with proven clinical applicability as prognostic and/or predictive factors in CRC and also to establish a feasible algorithm of molecular testing, as routine practice, in the personalized, multidisciplinary approach of colorectal cancer patients in our country.

Despite the revolu¬tion that occurred in the field of molecular marker research, only Serum CEA, Immunohistochemical analysis of mismatch repair proteins and PCR testing for KRAS and BRAF mutations have confirmed their clinical utility in the management of colorectal cancer. Their implementation in the current practice should partially resolve some of the controversies related to this heterogenic pathology, in matters of prognosis in different TNM stages, stage II patient risk stratification, diagnosis of hereditary CRC and likelihood of benefit from anti EGFR therapy in metastatic disease.

The proposed algorithms of molecular testing are very useful but still imperfect and require further validation and constant optimization.

## Introduction

Colorectal cancer (CRC) is one of the most frequent malignancies in Romania, with an incidence of 23,57/100000, more than 4000 new cases being diagnosed annually, representing 8% of all cancers. CRC is the third most frequent malignancy in our country, both in men and women after bronchopulmonary and gastric cancer and breast and uterine cancer, respectively. 90% of CRC are diagnosed after 50 years of age. The risk of developing CRC after 50 years is 5%, death related to CRC occurring in approximately 2.5% [**1**]. In the absence of a national screening program in CRC, most cases are diagnosed in advanced stages – 25% are presented as emergencies (obstruction, perforation, hemorrhage) and only a small percent are diagnosed in Stage I.

 Significant advances have been made in the treatment and outcome of CRC over the last decade, due to advanced screening tests, standardized surgical procedures and the advent of newer drugs, including biologic agents. The development of new agents has been facilitated by our improved understanding of the molecular pathways involved in the development and progression of CRC. 

 Most patients presenting with stage I, II, or III disease (75%) can be treated with surgery alone, or in combination with chemotherapy (high risk stage II, stage III) and radiotherapy (for rectal cancer), and have a 5-year survival rate of 93.2%, 82.5% and 59.5% respectively, compared with only 8.1% survival rate in stage IV disease [**2**]. The clinical management of CRC is currently based on clinicopathological factors such as TNM staging introduced by the American Joint Committee on Cancer (AJCC) and the International Union Against Cancer (UICC), histology, tumor margin status and performance status. Additional histopathological features such as lymphovascular inva-sion, peritoneal involvement and degree of differentiation along with clinical features such as obstruction and perfora¬tion at the time of diagnosis are currently used to better define the poor prognostic subsets of patients [**3**]. Although it has brought great benefit, the multimodal treatment based on TNM staging remains imperfect. Individual patients with same stage tumors may have different long-term prognosis and response to therapy. Stages IIB and IIC (AJCC) have lower survival rates than the more advanced Stage IIIA [**4**]. TNM staging alone cannot identify the high-risk group of 20–30% of patients with locally restricted stage II (UICC) colon cancer who will suffer from disease relapse. This group of patients may actually benefit from adjuvant therapy in addition to surgical treatment, even though chemotherapy is not usually recommended, according to current guidelines [**5**]. 

 Despite the extensive use of chemotherapy, mechanisms involved in clinical response remain elusive. A significant proportion of patients receiving various protocols of chemotherapy in metastatic disease do not derive any advantage. It becomes essential to identify subgroups of patients who may benefit from specific, targeted agents used in combinations such as FOLFOX (5 Fluorouracil + Leucovorin + Oxaliplatin), XELOX (Capecitabine + Oxaliplatin), FOLFIRI (Irinotecan + 5 F U + Leucovorin) or biological agents such as Epidermal Growth Factor Receptor Inhibitors (Cetuximab, Panitumumab) and Vascular Endothelial Growth Factor (Bevacizumab), in order to avoid a potentially toxic over treatment and an unprofitable financial burden for the health care system [**6**]. 

 Thus, research and validation of new prognostic factors, defined as any parameter evaluated at diagnosis (or surgery), which is associated with treatment outcome (disease free interval, survival, local control) and predictive factors, defined as any parameter which evaluates the response or lack of response to specific treatment is the key towards an optimized and personalized treatment of CRC [**7**]. Clinical research of molecular biology in the last two decades confirmed CRC as a heterogeneous complex of diseases through the accumulation of distinctive genetic and/or epigenetic alterations. It is believed that CRC may arise from at least three interlinked mechanisms: Chromosomal Instability (CIN), CpG island methylation phenotype (CIMP) and Microsatellite Instability (MSI) [**8**,**9**]. The current thinking behind CRC tumor genesis led to the development of numerous potential prognostic and predictive biomarkers. Unlike other types of cancer, with the exception of KRAS mutation, few of the studied markers have entered the clinical management of colorectal cancer so far, due to inconclusive studies or lack of current clinical applicability although they are widespread available in molecular biology laboratories [**10**]. The Aim of this review is to identify and discuss the molecular markers with proven clinical applicability such as prognostic and/or predictive factors in CRC and also to establish a feasible algorithm of molecular testing, as routine practice, in the personalized, multidisciplinary approach of colorectal cancer patients in our country. 

 CEA 

Serum Carcinoembryonic Antigen (CEA) is a complex serum glycoprotein produced by 90% of CRCs that functions as a homotypic intercellular adhesion molecule that promotes the aggregation of human CRC cells. CEA may facilitate metastasis of CRC cells to the liver and lung [**11**]. An ideal way to assess tumor status would be a serum marker. Serum markers allow a minimally invasive method of CRC screening that could easily be integrated into regular health checks. Currently, CEA is the only serum biomarker in a widespread clinical use for CRC that can be quantitatively measured. CEA is a normal cell product that is overexpressed by adenocarcinomas, primarily of the colon, rectum, breast and lung [**12**]. Smokers have a higher circulating CEA concentration (13.6%) than non-smokers (1.8%), but there are no significant effects of age, sex, or ethnic group on the normal range. In metastatic disease, 30% of patients may have normal CEA. Moderate to significant elevations of serum CEA can be observed in a variety of chronic and acute inflammatory diseases, including alcoholic cirrhosis, cholelithiasis, obstructive jaundice, cholangitis, liver abscess, emphysema, bronchitis, gastric ulcer, gastritis, diverticulitis, diabetes and collagen vascular diseases [**13**]. 

Current ASCO guidelines recommend preoperative determination of CEA and also regular (every 2–3 months) CEA monitoring for 2–3 years follow-up in patients with stage II and stage III CRC who might be amenable for metastatic surgery upon recurrence and also in monitoring patients with advanced disease, receiving palliative chemotherapy [**14**]. However, because of its lack of sensitivity in the early stages of CRC, CEA testing alone is an unsuitable modality for population screening. Wild et al found that the combination of six biomarkers had a sensitivity equal to fecal immunohistochemical testing for the early detection of colorectal cancer, CEA showing the best sensitivity at 95%, with a specificity of 43.9% followed by seprase (42.4% sensitivity), CYFRA 21-1 (35.5%), osteopontin (30.2%), ferritin (23.9%) and anti-p53 (20.0%) [**15**]. In some studies, the presence of elevated preoperative CEA (>5 ng/ml) correlates with poor prognosis and reduced overall survival in Stage II colon cancer [**16**,**17**]. CEA could therefore be used in identifying high-risk Stage II patients who might benefit from adjuvant therapy [**3**]. 

In a previous study, we found that a positive rate of serum CEA was significantly higher in advanced stages of disease, also in poorly differentiated colorectal cancer than in well and moderately-differentiated types [**18**]. CEA is also associated with Microsatellite Instability of CRC, as shown in another study, in which Stage III cancers with MSI had an elevated preCEA more often than those without MSI (25%vs. 0%; p = 0.026) [**19**]. A failure of the CEA to return to normal levels after surgical resection is indicative of inadequate resection (elevated plasma CEA levels should return to normal within 4 to 6 weeks), or of occult systemic disease, as reported for elevated postoperative CEA levels [**20**]. Other studies found that CEA levels above zero were indeed more predictive than any level within the normal range or above. Thus, absent CEA after surgery appears to confer a favorable prognosis. In 2006, Park et al. [**19**] investigated the role of serum CEA level in predicting the response to preoperative radiotherapy in rectal cancer. CEA level (>5 ng/mL) was significantly associated with both node infiltration and poor response to radiotherapy. In 562 patients with non-metastatic rectal cancer, who received pRCT and underwent total mesorectal excision, Das et al. [**21**] confirmed that CEA level (>0.25 ng/mL) predicted tumor downstaging along with circumferential extent and distance of tumor from the anal verge. Moreover, Perez et al. [**22**] found that a post-radiotherapy CEA level <5 ng/mL was a favorable prognostic factor for rectal cancer and was associated with increased rates of earlier disease staging and complete tumor regression. 

 Serial CEA measurements can detect recurrent CRC with a sensitivity of roughly 80% and speciﬁcity of roughly 70%, and can provide a lead time of approximately 5 months [**23**]. However, the test often adds little useful information in the follow-up of CRC. Most evaluations of treatment success or failure will be made on the basis of imaging studies, such as CT or MRI scans. In general, CT or MRI should trump CEA changes, and a patient should not have treatment changed on the basis of a CEA alone, unless the patient has a relatively uncommon circumstance, such as predominantly peritoneal metastases, in which CT or MRI evaluation is extremely limited. If the CT shows clear improvement or worsening and the CEA result disagrees, the CEA result is going to be (or at least, should be) ignored. 

 Microsatellite instability 

Research in molecular biology divided the pathogenesis of colorectal cancers into two pathways: (1) the chromosomal instability pathway (CIN) with accumulation of chromosomal abnormalities; (2) the microsatellite instability pathway (MSI). 

About 85% of all colorectal cancers fall into the CIN category, with the vast majority occurring as sporadic, non-hereditary manifestations. The remaining 15% of MSI-related cancers break down into 10%–12% sporadic tumors and 3%–5% hereditary cancers (HNPCC). 

Microsatellites (MS) are stretches of DNA in which short sequences (usually 1–5 nucleotides long) are repeated. Given their repetitive nature they are liable for errors that can occur during DNA replication. Mismatch repair genes (MMR) play a critical role in the identification and correction of these errors. Failure of the mismatch repair apparatus leads to persistence of errors and an alteration in the length of a microsatellite sequence, a process described as microsatellite instability. Persistence of such errors in the critical areas of genes responsible for cell growth regulation leads to frameshift mutations with loss of the normal function of these genes and the promotion of tumorgenesis. MMR deficiency may result from the inheritance of a MMR gene mutation, somatic (noninherited) MMR gene alterations, epigenetic suppression of MMR gene expression or a combination of these factors [**25**]. 

The DNA mismatch repair system requires the cooperation of many genes including MLH1, MSH2, MSH3, MSH6 and PMS2. Germline mutations in one of the MMR genes are responsible for a genetic predisposition to CRC, known as Lynch syndrome, previously referred to as hereditary nonpolyposis colorectal cancer (HNPCC). In sporadic CRCs, MSI appears to arise owing to an epigenetic phenomenon, namely silencing of the MLH1 gene by hypermethylation of CpG islands in its promoter region [**24**]. 

**Fig. 1:**
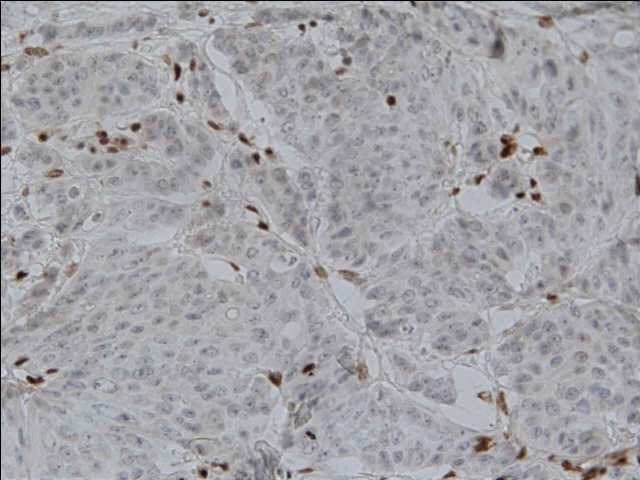
Immunohistochemical (IHC) analysis of mismatch repair proteins Negative staining (abnormal) for MLH1

 MSI testing can be performed either by PCR amplification (1) of extracted DNA (from a tumor sample, as well as from control normal tissue) and by IHC analysis of MMR proteins (2). Although both these tests can identify MSI tumors with great sensibility and specificity, they cannot differentiate properly the cause of the MMR deficiency required for the differential diagnosis of sporadic and inherited CRC. Further specialized testing include germline mutation analysis of the affected MMR genes, Methylation-specific PCR and testing for the BRAF V600E mutation. 

 (1)The PCR products can be separated electrophoretically and differences in fragment sizes from tumor-derived DNA versus DNA from normal tissue are scored, leading to an assessment of instability. At first, there was a great variability of the microsatellite loci selected. A National Cancer Institute (NCI)-sponsored workshop in 1998 recommended a panel of five microsatellite markers, known as the Bethesda markers, which consisted of two mononucleotide (BAT-25 and BAT-26) and three dinucleotide (D5S346, D2S123 and D17S250) repeats [**26**]. Samples with instability in two or more of these markers were defined as MSI-H (for high-frequency MSI), whereas those with one unstable marker were designated MSI-L (for low-frequency MSI). Samples with no instability in any of the markers were considered to be microsatellite stable (MSS). In 2004, the revised Bethesda guidelines included another panel of five quasimonomorphic mononucleotide repeats (BAT-25, BAT-26, NR-21, NR-22 and NR-24) [**25**]. There are no conclusive molecular or clinicopathological differences between MSL-L and MSS tumors, as studies reported [**26**], thus from the clinician point of view, only positive testing for MSI-H tumors should be considered. 

 (2)IHC analysis of MMR proteins, widely available as part of the routine services in general pathology, has become a popular alternative to detect MSI in the clinical setting. DNA mismatch repair proteins are normally expressed in normal human tissues, however in MSI-CRC a complete loss of protein expression of at least 1 of the MMR genes appears. This loss of protein expression is pinpointed by the absence of immunohistostaining using a panel of 4 antibodies to the protein product of the 4 genes most frequently involved in MSI-CRC: MLH1, MSH2, MSH6, PMS2 [**27**] (**Fig. 1**). 

If all 4 proteins are expressed, then we have a MSS phenotype. The interpretation of the results should follow a good understanding of the interaction of MMR proteins as MLH1 binds to PMS2 to the site of DNA repair and the same happens with MSH2 and MSH6 [**28**].

If the MLH1 gene is affected, either by germline mutation or CpG hypermethylation, then normal expression of MLH1, and also PMS2 will be lost, with preserved MSH2 and MSH6. If MSH2 gene is affected by germline mutation, then MSH2 and MSH6 expression will be lost. In contrast, in the rare cases of germline mutations of either PMS2 or MSH6, only the protein affected is lost [**29**]. 

In a comparative study, the predictive value of normal IHC for an MSS/MSI-L phenotype was 96.7%, and the predictive value of abnormal IHC was 100% for an MSI-H phenotype , resulting in a sensibility of 94% and specificity of 100% in identifying MSI-H tumors [**30**], similar with MSI PCR testing. As studies confirmed, MSI testing and IHC appear to be almost equivalent strategies for identifying subjects with affected MMR genes – For the clinician, MSI-H phenotype at MSI testing and dMMR at immuhistostaining should be equivalent and considered as MSI-H [**31**]. However, identifying a MSI-H phenotype in CRC by MSI-PCR or by IHC is not enough to differentiate between an inherited and a sporadic lesion (See the diagnosis of Lynch Syndrome below). Both inherited and sporadic MSI-H tumors have very distinct clinical characteristics: proximal tumor location, female gender dominance, mucinous histology, lymphatic infiltration, high number of peritumoral lymph nodes, undifferentiated histology [**32**]. 

The MSI phenotype has three major clinical implications. Besides the role in the diagnosis of Lynch Syndrome, which implies an individually tailored, and familial surveillance program and prophylactic treatment, studies suggest that MSI has also prognostic and predictive value in the management of colorectal cancer patients. 

**Fig. 2:**
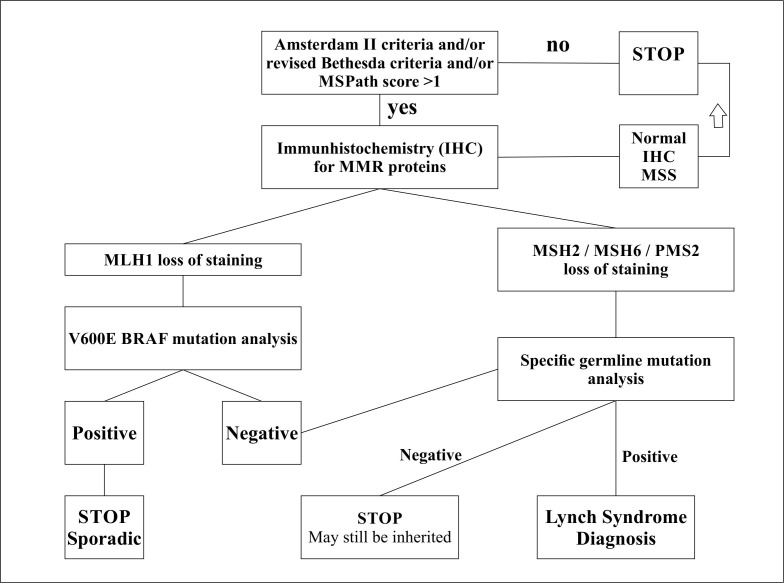
Suggested algorithm for molecular and genetic testing for Lynch syndrome IHC: Immunohistochemistry; MSI: Microsatellite instability; MMR: Mismatch Repair; MSS: microsatellite stable

 Lynch Syndrome is a familial autosomal dominant condition characterized by a germline mutation in one of the MMR genes (90% - MLH1 and MLH2, 5-10% MSH6, PMS2) [**33**-**35**]. The 2-hit hypothesis of tumorigenesis applies to Lynch syndrome, where the first hit is represented by the germline mutation in 1 copy of 1 MMR and the “second hit is a somatic inactivation of the wild type allele”. The inherited defect results in a lifetime risk of 80% of developing early-onset and even multiple CRC in the setting of relatively few polyps (mean age, 45 years). The syndrome has an incidence of approximately 1:1000 in the general population and accounts for 3–5% of CRCs. These patients also have an increased risk of extracolonic malignancies; endometrial cancer is the most common extracolonic cancer (40-60%) followed by gastric, small intestinal, hepatic, pancreaticobiliary, ovarian, ureteral, and brain tumors [**36**,**37**]. The terms Lynch syndrome and hereditary nonpolyposis colorectal cancer have largely been used interchangeably over the years. The definition of HNPCC is primarily based on family history. Without knowledge of the family history, a diagnosis of HNPCC cannot be made. Lynch syndrome, on the other hand, is defined by the documentation of inherited inactivating mutations in the DNA MMR system and thus, without a molecular genetic testing of DNA from peripheral blood or normal tissue of the MMR genes to identify a germline mutation, a diagnosis of Lynch syndrome cannot be made [**38**,**39**]. However, full gene sequencing in all CRC patients is highly inefficient. Various versions of sequential testing algorithms have been proposed for the diagnosis of Lynch Syndrome in which family history, clinical and pathological criteria correlated with IHC or PCR MSI testing, narrow considerably the number of patients that should undergo genetic testing, and in many cases, can even eliminate the need of further testing [**40**-**42**] (**Fig. 2**). 

The first major clinical criteria were set by the International Collaborative Group on Hereditary Non-Polyposis Colorectal Cancer meeting held in Amsterdam in 1990 [**43**]. These criteria (now known as Amsterdam criteria I) which addressed only personal and family histories of CRC were set to be the basis of the diagnosis of HNPCC. At the 1998 group meeting, these criteria were updated to include also the known extracolonic tumors associated with Lynch Syndrome (now known as Amsterdam criteria II – **Table 1**) [**44**].

**Table 1:**
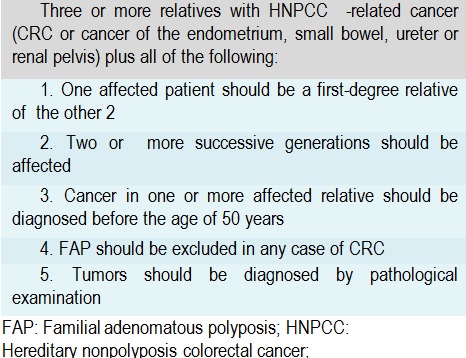
Amsterdam II criteria

Since then, the growing understanding of the molecular basis of Lynch Syndrome permitted numerous studies to demonstrate that the sensibility of the Amsterdam II criteria is quite low (40%) [**45**,**46**]. Possible explanations for this lack of reliability include small family size, the physicians’ unfamiliarity with the syndrome, lack of documentation or reduced penetrance of the MMR gene mutation in the family. Also, the specificity of the Amsterdam II criteria is low. 50% of patients whose families fulfill the Amsterdam criteria fail to demonstrate MSI on tumor testing or germline mutations in any of the MMR genes. Such families are unlikely to have Lynch syndrome and may represent an unknown hereditary colon cancer syndrome. These families have an increased risk of developing CRC compared with the general population, but compared with Lynch syndrome patients, the risks are lower, with an older age of onset and no increased risk of extracolonic cancers [**47**].

 The Bethesda Guidelines formulated in 1996 and revised in 2004 [**48**,**49**] incorporate broader age and family history criteria, but also include the unique histopathological features associated with Lynch tumors (tumor-infiltrating lymphocytes, signet ring cells, mucinous and poorly differentiated histology) as a fifth criteria (**Table 2 **). The novelty of the Bethesda Guidelines was the introducing of MSI testing in colorectal tumors that fulfill the criteria as a mandatory step in the diagnosis of Lynch Syndrome. 

**Table 2:**
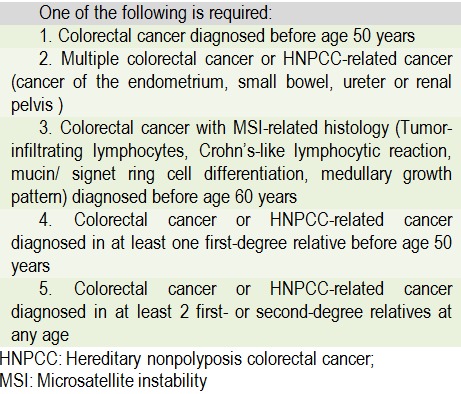
Revised Bethesda Guidelines

In 2007, Jenkins et al. published the MsPath model that used easily assessable clinicopathological characteristics to identify colorectal cancers with microsatellite instability (MSI-H), presenting in patients younger than 60 years, the age group most likely to be associated with LS, while ruling out colorectal cancers that are highly unlikely to be MSI-H. This model considers six clinicopathological features (with the corresponding coefficient): age at diagnosis (<50-year -0.7), anatomical site (cecum, ascending or transverse colon -1.6), histologic type (mucinous, signet ring, or undifferentiated -1.1), grade (poorly differentiated -0.6), Crohn-like reaction (present -0.5), and tumor-infiltrating lymphocytes (TILs) (present -2.1). Authors have recommended a cutoff MsPath score of 1.0 to maximize the specificity while maintaining a high sensitivity, because it is important not to miss MSI-H cases [50]. Studies tried to evaluate the practical validity of revised Bethesda criteria, MSI histology, and MsPath model [9] for detection of MSI-H phenotype in CRC and found a sensibility of 79% (confidence interval [CI], 54% to 93%), and a specificity of 77% (CI, 68.7% to 83.7%) for the Bethesda guidelines. MSPATH score achieved 93% sensitivity and 55% specificity [**51**,**52**].

As seen, both familial and clinicopathological criteria were insufficiently accurate for routine use as a stand-alone screen for patients with CRC warranting MMR mutation testing but proved useful tools in selecting patients who should undergo MSI testing either by PCR or IHC, as an intermediate step in the diagnosis of Lynch Syndrome. Current debate revolves around whether all colorectal tumors should undergo MSI testing or only those that fulfill specific clinical criteria.

Our approach is to apply the Amsterdam II and the revised Bethesda criteria and also the MsPath model (cutoff >1) to all CRC patients. The fulfillment of either one is an indication for IHC-MMR analysis (Fig. 2). Patients with MSH2, MSH6 or PMS2 protein loss at imunohistostaining are considered highly suspects for Lynch Syndrome and referred for specific germline mutation testing and genetic counseling. IHC showing loss of MLH1 expression could be seen in either sporadic MSI-CRC or Lynch syndrome. In these cases, an additional layer of testing may be performed on the tumor before proceeding to germline analysis. These tests include MLH1 promoter methylation and/or BRAF mutation analysis. Given the technical difficulty related to methylation analysis, testing for BRAF mutations is more commonly performed. The V600E hotspot mutation in exon 15 of the BRAF gene, a member of the RAF family of kinases, has demonstrated utility in distinguishing whether the loss of MLH1 staining is due to somatic hypermethylation of MLH1 or a germline mutation [**53**,**54**]. Deng et al. found that this specific BRAF mutation occurred in 87% of sporadic tumors with hypermethylated MLH1, whereas it was not present in any MSI tumor with a germline MLH1 mutation [**55**], hence, a ‘‘positive’’ BRAF mutation virtually excludes the possibility of Lynch syndrome. If the BRAF mutation is negative, then germline mutation analysis should be performed (Fig. 2).

The demonstration of a germline mutation is the gold standard for the diagnosis of Lynch syndrome. Germline testing is performed on DNA isolated from peripheral blood mononuclear cells. More than 90% of Lynch syndrome cases that have been genetically characterized show germline mutations in MSH2 or MLH1 [**55**]. However, the selection of which gene to test is dependent on the abnormality identified on the initial screening test. Once the diagnosis of Lynch syndrome has been established, patient genetic counseling and discussion of cancer risks and of the pros and cons of surgical options is crucial. An intensive surveillance program should be initiated and close family members can proceed with testing for the identified specific germline mutation. Current guidelines, both in Europe and in the USA, recommend colonoscopy starting by the age of 20–25 years every 2 years until the age of 40 years, at which point examinations should be performed annually. When a diagnosis of CRC is made in a MMR gene mutation carrier, subtotal colectomy with ileorectal anastomosis is recommended, owing to the 16% risk for developing a second primary CRC within 10 years [**56**]. Endometrial cancer in Lynch syndrome is usually recognized early (stage I), and the overall 5‑year survival rate is 88%. Annual transvaginal ultrasound and endometrial aspiration biopsies beginning between age of 25 and 35 years can be considered. 

Increased risks of ovarian cancer have also led some to propose annual transvaginal ultrasound with CA-125 testing beginning at the age of 30 years. Risk-reducing hysterectomy and bilateral salpingo-oophorectomy should also be presented as an option when childbearing is completed [**57**]. The lifetime risks of developing stomach, ureter, renal pelvis, small bowel, bile duct and brain cancers are lower. Surveillance with upper endoscopy is offered to all Lynch syndrome patients every 2 years, starting from the age of 30–35 years. Annual renal ultrasound, urinalysis and urine cytology can be considered in families with a history of urinary tract tumors [**58**].

Microsatellite instability as a Prognostic Marker 

 One of the key clinical features of MSI-CRC is their good prognosis and non-aggressive biology in spite of a commonly found undifferentiated histology. One of the consequences of this behavior is that the prevalence of MSI-H colorectal cancers is higher in earlier compared with later tumor stages. Specifically, MSI-H tumors account for up to 22% of all stage II colon cancers, but only for 12% and about 5% of colorectal cancers diagnosed as stage III and IV, respectively [**26**]. In addition, MSI-H cancers are preferably right-sided with a decreasing prevalence of the MMR-D phenotype from proximal to distal locations, so that only around 4% of rectal cancers are MSI-H.

 In 2000, Gryfe et al. reported a population based study in which MSI was associated with a significant survival advantage, independent of all standard prognostic factors, including tumor stage (hazard ratio [HR]: 0.42;p < 0.001). Furthermore, regardless of the depth of tumor invasion, MSI CRC had a decreased likelihood of metastasizing to regional lymph nodes (HR: 0.33; p < 0.001) or to distant organs (HR: 0.49; p = 0.02) [**59**]. These results have been confirmed by many subsequent studies, including a meta-analysis by Popat et al. of 32 studies with more than 7000 CRC patients, approximately 1300 of whom demonstrated MSI. The HR estimate for overall survival associated with MSI was 0.65 [**60**] More recent results from large individual randomized adjuvant trials (PETACC-3 [**61**] and QUASAR [**62**]) clearly validated the prognostic implication of microsatellite instability with hazard ratios for relapse-free survival (RFS), disease-free survival (DFS), and overall survival between 0.16 and 0.70. More interesting is the fact that both trials trial demonstrated a very strong prognostic effect of MSI-H compared with non–MSI-H for stage II colon cancers but only an attenuated prognostic effect in stage III tumors.

 In conclusion, the MSI phenotype has unanimously been recognized as a marker of good prognosis. The risk of recurrence of a MSI-H stage II colon cancer is in the range of 3%–6% within the first 3 years, even without any adjuvant therapy [**62**].

Microsatellite instability as a Predictive Marker

 The relationship between MSI status and response to chemotherapy has been more controversial. Although early nonrandomized studies indicated a potentially higher activity of 5-FU–based chemotherapy in MSI-H than in MSS CRC, data from randomized clinical trials consistently suggested quite the contrary: patients with tumors exhibiting the MSI-H phenotype did not derive any benefit from 5-FU–based adjuvant chemotherapy, and might even have a detrimental effect. [**62**]. 

 Special attention in this context received Sargent et al. [**63**] who pooled the individual patient data from five randomized adjuvant trials which compared a 5-FU–based adjuvant chemotherapy against surgery alone in stage II and stage III colon cancer. There was a statistically significant detriment in overall survival in stage II MSI-H tumors treated with 5-FU–based adjuvant chemotherapy compared to the untreated cohort (HR 2.95, 95% CI 1.02–8.54, P00.04). This negative effect of adjuvant therapy was not found in stage III colon cancers. Therefore, the overall consensus has been that MSI is a predictor of nonresponse to 5-FU in stage II colon cancers. The prognostic and predictive value of MSI has notable implications for clinical practice. The mainstay of treatment for stages II and III CRC is surgical resection, with preoperative radiotherapy in rectal cancers. In stage III colon cancer, postsurgical adjuvant fluoropyrimidine-based chemotherapy has been the standard of care since the 1980s when 2 landmark studies demonstrated that fluorouracil plus levamisole reduced mortality by approximately 30% in patients with lymph node-positive (stage III) cancer [**63**,**64**].

**Fig. 3:**
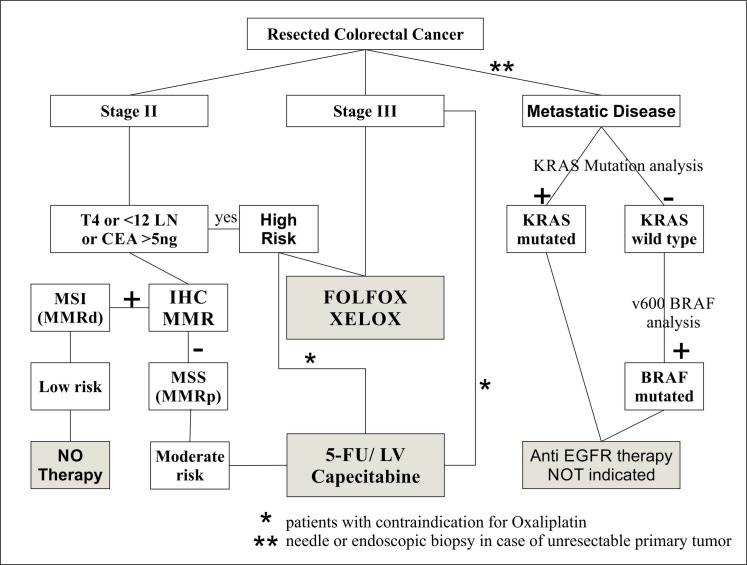
Proposed decision algorithm for adjuvant therapy in colon cancer LN: Lymph nodes; CEA: Serum Carcinoembryonic Antigen; IHC: Immunohistochemistry; MSI: Microsatellite instability; MMR: Mismatch Repair; MSS: microsatellite stable; FOLFOX: 5-Fluoro-uracil (5-FU) +Leucovorin (LV) + Oxaliplatin; XELOX: Capecitabine + Oxaliplatin; Anti EGFR therapy - Epidermal Growth Factor Receptor Inhibitors (Cetuximab, Panitumumab)

 In 2004, the MOSAIC trial showed that the addition of oxaliplatin to 5-fluorouracil and leucovorin (FOLFOX) reduced relapse by comparison with fluorouracil and leucovorin alone with hazard ratio (HR) 0.76 (95% CI, 0.62-0.92) [**65**]. Based upon the MOSAIC trial, 6 months of combination chemotherapy with the FOLFOX regimen is now the recommended adjuvant treatment for patients with stage III colon cancer, with no concern of the MSI status [**66**] (**Fig. 3**).

The use of adjuvant chemotherapy in patients with curatively resected stage II remains controversial, since not all patients are likely to receive any benefit. Current consensus guidelines require a risk stratification of patients based upon the presence of clinical, pathological and molecular risk factors. The high-risk features included in the current guidelines of the National Comprehensive Cancer Network (NCCN) are: bowel obstruction, grade 3-4 histology, T3 tumors with localized perforation, lymphatic or vascular Invasion, T4 Tumors, close, indeterminate or positive margins, less than 12 lymph nodes examined [**67**]. However, not all of them were confirmed by subsequent studies. In fact, in the QUASAR study, only T4 and inadequate lymph node sampling (<12) were confirmed as independent poor prognostic factors, while high grade of tumor, paradoxically, was a positive prognostic factor (perhaps due to its strong association with mismatch repair protein deficiency) [**62**]. 

 In addition, as seen above, preoperative CEA>5ng/dl can be regarded as a high risk feature in stage II patients [**6**]. In Fig. 3, we present our decision algorithm in the approach toward adjuvant therapy in all CRC stages. We consider high risk Stage II, patients presenting one of the following: T4, less than 12 lymph nodes examined and CEA>5ng/ml. These patients should follow the same postoperative protocols as in stage III CRC, as highlighted in the MOSAIC trial (High risk Stage II patients’ disease-free survival at 5 years was 82.1% with the use of FOLFOX by comparison with 74.9% with fluorouracil plus leucovorin). The same trial found that Stage II with none of the high risk features received no recurrence or survival benefit with the use of FOLFOX by comparison with fluorouracil plus leucovorin alone so these patients should be spared from the toxicity of oxaliplatin treatment [**65**]. These patients are further stratified into low risk (those who exhibit MSI-H/MMR-D phenotype) with good prognosis and no need for postoperative chemotherapy and intermediate risk (MSS phenotype), that should be considered for 5FU based treatment.

 KRAS, BRAF and Anti EGFR therapy

 KRAS is one of the most studied proto-oncogenes. A review of large trials reveals that approximately 30%-50% of metastatic CRC tumors harbor KRAS mutations. The mutations predominantly involve codons 12 and 13 of the KRAS gene and appear in an equal distribution at all sites of the colorectum [**68**]. Ras proteins occupy a key position in the Epidermal Growth Factor Receptor (EGFR) signaling pathway, which is involved in cell differentiation, proliferation, and angiogenesis.

 The introduction of EGFR-targeted monoclonal antibodies (cetuximab and panitumumab) in the treatment of metastatic colorectal cancer transformed KRAS mutation testing from a fundamental research to an important predictive marker with proven clinical applicability.

 Combination cytotoxic regimens such as FOLFOX and FOLFIRI have typically been the chemotherapy backbones for first line treatment for metastatic colorectal cancer. The adding of Cetuximab to FOLFOX or FOLFIRI was studied into two large trials – OPUS [**69**], CRYSTAL [**70**], in which tumors were also evaluated for KRAS status. Studies found significant improvement in response rate (RR) and progression-free survival (PFS) in both associations (RR, 61% vs. 37%; P = .01; hazard ratio [HR] for progression, 0.57; P = .016), but only with patients with wild type KRAS. Patients with a KRAS mutation had a significant worsening in outcome with the addition of Cetuximab to both FOLFOX and FOLFIRI, demonstrating that mutated KRAS is a negative predictive factor for anti EGFR therapy. The same negative predictive value of KRAS mutation status was found on Cetuximab or Panitumumab monotherapy as second-line treatment of refractory metastatic CRC [**72**]. In February 2009, ASCO published a provisional clinical opinion unequivocally recommending KRAS gene testing in patients with metastatic CRC to select patients appropriate for therapy with Cetuximab or Panitumumab [**71**]. The National Comprehensive Cancer Network (NCCN) has also added a recommendation for KRAS testing to their revised 2009 Clinical Practice Guidelines for Colon Cancer, stating that patients with known KRAS mutations should not be treated with Cetuximab or Panitumumab, either alone or in combination with other agents [**67**]. Knowing the mutational status of a patient’s tumor before prescribing an EGFR-targeting MoAbs must now be considered the standard of care in metastatic CRC. At present, there are no clinical decisions that are impacted by KRAS mutation status in patients with stage I, II, or III colorectal cancer (Fig. 3). 

 Currently, KRAS mutations are identified by widely available PCR kits applied on formalin-fixed, paraffin-embedded tissue specimens from resected primary tumors or needle or endoscopic biopsies in unresectable metastatic CRC. In addition to the role in differentiating between sporadic and hereditary MSI-CRC (discussed above), BRAF mutation was found to have poor prognostic and predictive value in metastatic CRC. BRAF mutations are found in 10% of CRC; recent studies found that KRAS and BRAF mutation occur in a mutually exclusive manner [**74**]. Cappuzzo et al. [**73**] showed that patients whose CRC harbored BRAF mutation showed no response to anti- EGFR therapy, and they also showed a negative trend for both time to progression and survival.

 In conclusion, BRAF V600E mutation PCR testing is indicated in CRCs that are negative for KRAS mutation, when the patient is being considered for anti-EGFR therapy (Fig. 3).

## Conclusions

Although it remains a controversial issue in oncology practice and should be interpreted with caution, on a case by case basis, in correlation with stage, degree of differentiation and MSI status, CEA is a useful prognostic biomarker in CRC, due to its validated clinical applicability, wide availability and cost effectiveness. Despite the revolu¬tion that occurred in the field of molecular marker research, only Serum CEA, Immunohistochemical analysis of mismatch repair proteins and PCR testing for KRAS and BRAF mutations have confirmed their clinical utility in the management of colorectal cancer. Their implementation into current practice should partially resolve some of the controversies related to this heterogenic pathology, in matters of prognosis in different TNM stages, stage II patient risk stratification, diagnosis of hereditary CRC and likelihood of benefit from 5-FU chemotherapy or anti EGFR therapy in metastatic disease. The proposed algorithms of molecular testing are very useful but still imperfect and require further validation and constant optimization; clinical judgement must be exercised in all cases. 

In order to fully exploit the potential of molecular markers towards patients’ benefit, CRC should be treated in a multidisciplinary team of surgeons, pathologists and oncologists, in which knowledge transfer and feedback between team members is essential.

 In the current environment in which cost-effectiveness in medicine is an appropriate concern, the promising new markers being researched should be incorporated into clinical practice only after their prognostic and/or predictive value was demonstrated by randomized clinical trials and providing they will impact decision-making in a patient’s care.

 ACKNOWLEDGEMENT: 

 This paper is supported by the Sectorial Operational Programme Human Resources Development (SOP HRD) 2007-2013, financed from the European Social Fund and by the Romanian Government under the contract number POSDRU/107/1.5/S/82839
